# Immune imprinting toward SARS-CoV-2 XBB: implications for vaccine strategy and variant risk assessment

**DOI:** 10.1038/s41392-025-02484-5

**Published:** 2025-11-13

**Authors:** Xiaoyun Yang, Guichang Li, Yuan Wang, Tao Song, Tingting Cui, Jingjing Luo, Siyun Chen, Junjie Cao, Jiaying Zhong, Nanshan Zhong, Zhuxiang Zhao, Zhongfang Wang

**Affiliations:** 1 State Key Laboratory of Respiratory Disease & National Clinical Research Center for Respiratory Disease, Guangzhou Institute of Respiratory Health, the First Affiliated Hospital of Guangzhou Medical University, Guangzhou Medical University, Guangzhou, China; 2 Guangzhou National Laboratory, Guangzhou, China; 3 Department of Clinical Laboratory, State Key Laboratory of Respiratory Disease and National Clinical Research Centre for Respiratory Disease, The First Affiliated Hospital of Guangzhou Medical University, Guangzhou Medical University, Guangzhou, Guangdong, China; 4 Department of Infectious Disease, Respiratory and Critical Care Medicine, Guangzhou First People’s Hospital, Guangzhou Medical University, Guangzhou, China

**Keywords:** Vaccines, Infectious diseases, Adaptive immunity

## Abstract

The immune imprinting against SARS-CoV-2 subvariants that dynamically evolves through sequential vaccination and infection has been rarely studied. Using antigenic cartography and neutralizing antibody (NAb) profiling, we demonstrate that prototype-targeting vaccination followed by Delta/early Omicron breakthrough infections maintained dominant wild-type (WT)-focused immunity. However, XBB.1.5-adapted vaccination after BA.5 outbreaks shifted immune imprinting toward XBB.1.9.1, altering the antigenic landscape. NAb analysis revealed progressive WT-specific immunity enhancement through three-dose vaccination followed by BA.5 breakthrough infection (GMT: I-I = 35, I-I-I = 72, I-I-I-B5 = 807), followed by sharp decline after XBB reinfection (GMT: I-I-I-B5-XBB = 231), confirming XBB’s antigenic divergence. To investigate the relationship between population immune dynamics and XBB infection risk following the BA.5/BF.7 wave, we analyzed the immune status of XBB breakthrough-infected and uninfected individuals from May to June 2023 (5–7 months post-wave). Utilizing an infection model calibrated to NAb titers against XBB.1.9.1, we estimated the 50% protective NAb titer against XBB infection to be 1:12.6. Retrospective analysis revealed that 80.3% of the population fell below this threshold in mid-2023, aligning with subsequent XBB resurgence. However, only 33.8% exhibited sub-protective JN.1 titers (<12.6) by August 2024, explaining the absence of JN.1-driven endemicity. This longitudinal study maps the immune imprint transitions from WT dominance to XBB adaptation, providing critical insights into vaccine strategy optimization and emerging variant risk assessment. The work highlights how iterative immune exposures reshape population protection landscapes against evolving coronaviruses.

## Introduction

From December 2022 to January 2023, over 80% of the Chinese population was infected with BA.5/BF.7 subvariants, establishing widespread population immunity against these strains.^1,2^ Subsequently, China experienced three to five additional infection waves. A surge of XBB.1.9.2 and XBB.1.5 infections emerged in late April 2023, signaling declining host immunity in some individuals to levels permitting XBB breakthrough. By the third week of June 2023, the XBB subvariant EG.5 accounted for 24.7% of cases and remained dominant until December 2023.^3^ JN.1 became the predominant strain in early 2024, maintaining dominance until August 2024,^4^ when it was gradually displaced by XDV and KP.3. Over nearly 5 years (by the time of the study conducted), SARS-CoV-2 has evolved from the ancestral strain to the more recent subvariants such as JN.1, KP.2/3, and XDV. The antibody landscape in populations is shaped by sequential antigen exposures (vaccination or infection), disease severity, and interactions with newly encountered antigenically related subvariants. The concept of antigenic distance, initially developed to quantify differences between influenza antigens,^5^ was later defined as the antigen-antibody reactivity gap between distinct viral variants.^6^ This metric has been widely adopted in SARS-CoV-2 research to assess cross-protection between subvariants by measuring antibody titers against ancestral and emerging strains. However, population immune imprints have grown increasingly complex due to heterogeneous vaccination histories, multiple infection/reinfection events, and divergent viral circulation patterns across countries.^7,8^ These variations in immune landscapes, driven by differences in primary exposure (e.g., ancestral virus vaccines vs. infection-primed immunity), critically influence vaccination strategies, including decisions on booster timing, target populations, and immunogen selection (e.g., monovalent vs. variant-adapted vaccines).

Another important factor influencing vaccination strategy is whether the level of neutralizing antibodies (NAbs) in the population remains protective against the circulating or potential emergent subvariants. Different waves of XBB subvariants emerging at different times have raised questions regarding when the population immunity will decline to a protective threshold level of various XBB subvariants and whether such a threshold plays a role in the longitudinal emergence of SARS-CoV-2 subvariants. Two main methods, including individual-based correlates and population-based correlates have been used for several licensed vaccines based on a protective threshold or minimum protective level.^9^ The individual-based correlate of protection (CoP) measures antibodies in vaccinated individuals prior to exposure to the pathogen and assesses the relationship between antibody levels and disease progression. It is crucial to estimate a threshold concentration above which individuals are likely to be protected. This approach has been applied to several diseases, including measles,^10^ meningococcal diseases,^11^ influenza,^12^ and hepatitis B virus,^13^ to evaluate vaccine efficacy and monitor population immunity. However, since the outbreak of SARS-CoV-2 in late December 2019, the protective threshold for different SARS-CoV-2 variants has rarely been studied. At the time when our study was being conducted, SARS-CoV-2 subvariants such as XDV, KP.1, and KP.3 were still circulating around the world. It remained urgent for policymakers to predict the risk of emerging infections and decide when it would be appropriate to implement a proper national vaccination plan.

Antigenic distance has been extensively characterized by multiple research groups. Notably, studies demonstrate that JN.1 and KP.3 exhibit substantial immune evasion not only from the ancestral strain but also from later subvariants, such as BA.5 and XBB.1.9.^14^ However, updating vaccine strains depends not solely on the antigenic distance between existing vaccines and circulating/potential variants but also on the population’s preexisting immune background. To determine a protective threshold of SARS-CoV-2 NAbs, we recruited a cohort of individuals with or without XBB infection from fever clinic attendees and measured the XBB-specific NAb titers (NT_50_). Using multivariable logistic regression analysis, we established a 50% protection threshold as the NT_50_ at which half of the cohort was protected from XBB infection.

Our findings reveal that sequential vaccination and breakthrough infections, which occurred in individuals with pre-existing immunity due to immune escape or immunity waning, progressively shifted immune imprinting from the prototype strain toward early Omicron subvariants. Crucially, XBB-targeted vaccination attenuated WT-directed immune dominance in vaccinated individuals with prior BA.5 infection. By analyzing NT_50_ against XB.1.9.1 and the XBB-infection status of over 900 plasma samples with a multivariable logistic regression model, we estimated the 50% protective NAb titer against XBB.1.9.1 to be 12.6. This study provides real-world evidence of dynamic immune imprinting shifts driven by heterogeneous vaccination and infection histories. These insights offer actionable guidance for optimizing the timing and strategy of future vaccine updates, emphasizing the need to balance antigenic distance metrics with population immunity landscapes.

## Results

### Vaccination-infection history imprinted stratified immunity dynamics

**Fig. 1:**
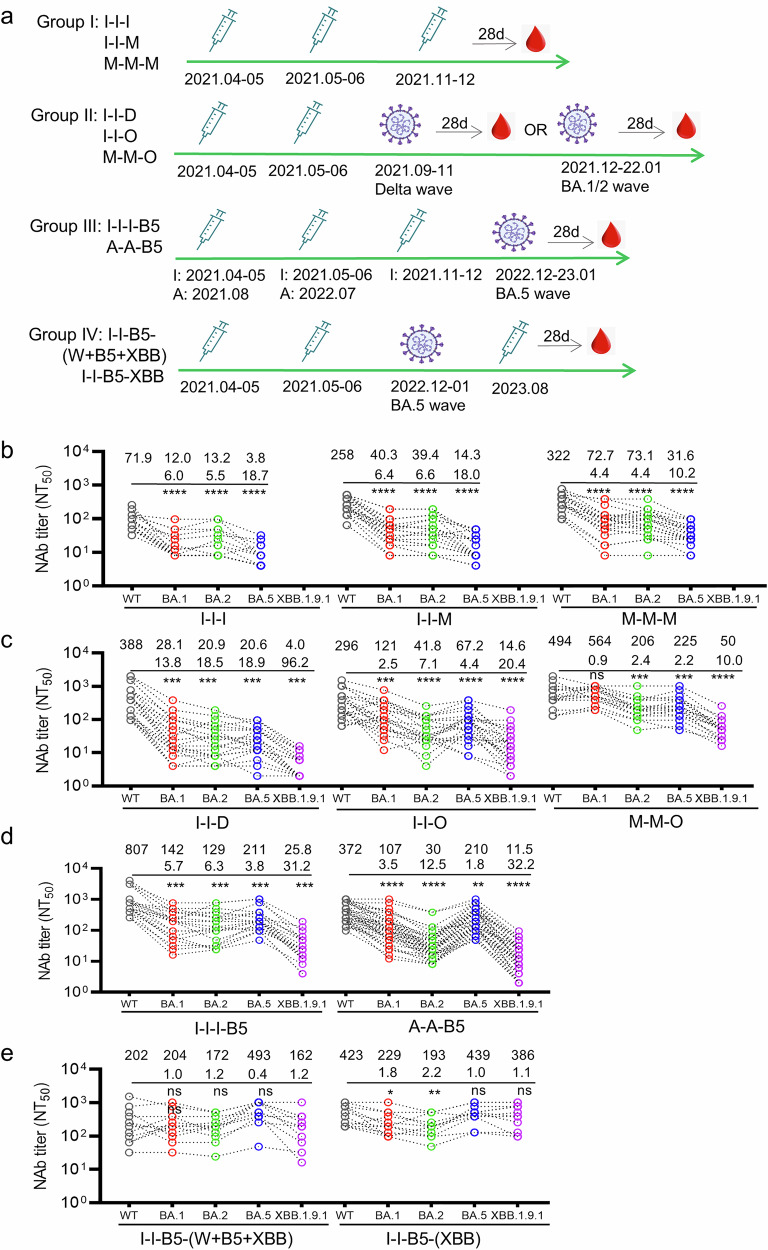
Neutralizing antibody responses against evolving SARS-CoV-2 variants post different vaccination and hybrid immunity. **a** Graphical representation of the study design indicating grouping, time points of vaccination, infection and sampling. **b** Neutralization activities of plasma against WT, BA.1, BA.2, BA.5 subvariants in individuals vaccinated with prototype vaccines (I-I-I: three doses inactivated viral vaccines, I-I-M: two doses inactivated viral vaccines boosted with mRNA vaccine, M-M-M: three doses of mRNA vaccines). Neutralization activities of plasma against WT, BA.1, BA.2, BA.5 and XBB.1.9.1 subvariants in individuals vaccinated with prototype vaccines followed by Delta (I-I-D) and early Omicron (I-I-O and M-M-O) breakthrough infection (**c**), in individuals vaccinated with prototype vaccines followed by BA.5 breakthrough infection (I-I-I-B5, A-A-B5) (**d**), and individuals vaccinated and BA.5 breakthrough infected, followed by XBB.1.5 targeting vaccination (I-I-B5-[W + B5 + XBB] and I-I-B5-XBB) (**e**). I-I-D: two inactivated vaccine shots followed by Delta breakthrough infection, I-I-O: two inactivated vaccine shots followed by BA.1/BA.2 infection, M-M-O: two mRNA vaccine shots followed by BA.1/BA.2 infection, I-I-I-B5: three inactivated vaccine shots followed by BA.5 infection, A-A-B5: two adenoviral vaccine shots followed by BA.5 infection; I-I-B5-XBB and I-I-B5-(W + B5 + XBB): two inactivated vaccine shots followed by BA.5 infection and then XBB-targeting or WT + BA.5 + XBB.1.5-targeting vaccination. The first line of numbers above dot plots are geometric mean titers, and the second line of numbers are fold-change of NAbs against WT over the corresponding subvariants. Student’s *t* test was performed for the statistics analysis between WT and other subvariants NAb titers with *p*-value as indicated (ns not significant, *: *p* < 0.05, **: *p* < 0.01, ***: *p* < 0.001, ****: *p* < 0.0001)

To investigate how vaccination and hybrid immunity affect neutralizing antibody responses against evolving SARS-CoV-2 variants, we measured plasma NAb titers against WT, BA.1, BA.2, BA.5, and XBB.1.9.1 subvariants at 28 days post-final immune exposure (vaccination or infection). The four groups of vaccination/hybrid immunity samples were collected according to timelines shown in Fig. 1a. Because the XBB.1.9.1 titers of most of the samples in the prototype vaccinated group were under the detection limit, which was 4 in the microneutralization assay, the data were not plotted in Fig. 1b. In primary vaccination groups (I-I-I, I-I-M, M-M-M), neutralization titers against different variants followed a consistent hierarchy: WT > BA.1 ≈ BA.2 > BA.5 (Fig. 1b). This immune change pattern persisted in individuals with prototype vaccination followed by Delta (I-I-D) or Omicron BA.1/BA.2 breakthrough infections (I-I-O, M-M-O) (Fig. 1c). Notably, BA.5 breakthrough infection cohorts (A-A-B5, I-I-I-B5) exhibited distinct profiles with the NAb titers following the pattern of WT > BA.5 > BA.1 ≈ BA.2, while XBB.1.9.1 demonstrated maximal immune evasion across all groups (Fig. 1d). Critically, XBB.1.9.1 consistently elicited the lowest NAb titers regardless of immune history before BA.5 exposure (Fig. 1b–d), underscoring the persistent antigenic seniority of the prototype strain in both vaccination-only regimens and early Omicron variant breakthrough infections preceding boosting. Interestingly, XBB boosting post-BA.5 infection paradoxically enhanced BA.5-specific responses to surpass WT-specific NAbs, and significantly increased XBB NT_50_ to that of similar levels of WT (Fig. 1e).

### Antigenic distance analysis of vaccination-infection profiles

**Fig. 2:**
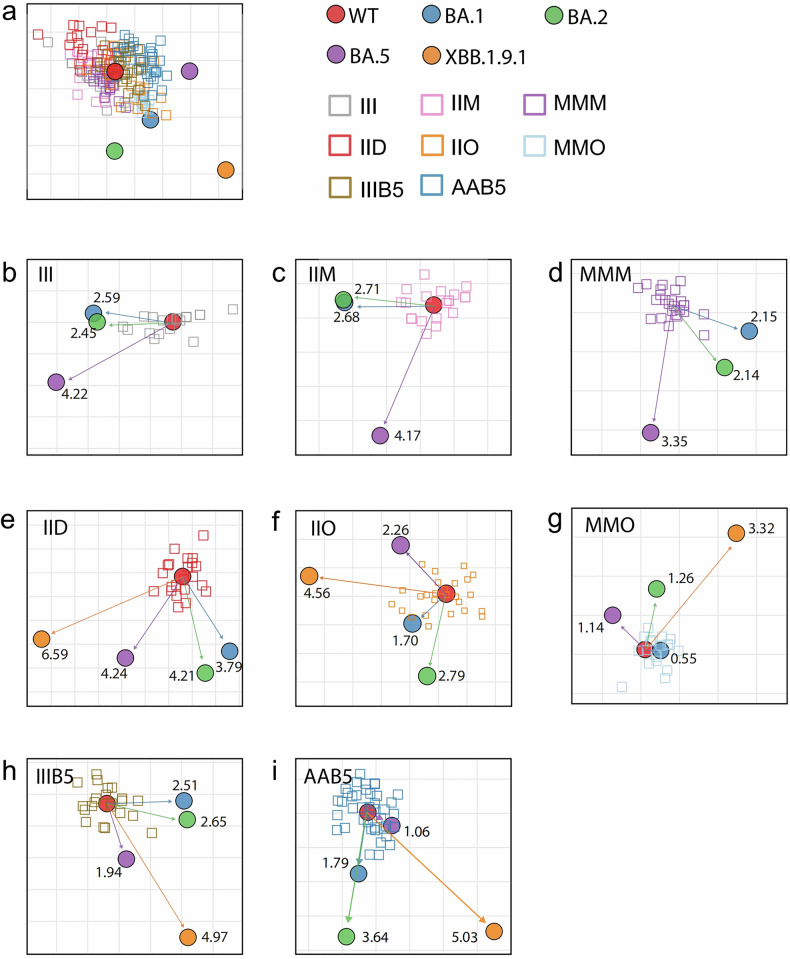
Antigenic cartography analysis of SARS-CoV-2 in vaccinated or vaccinated and breakthrough infected immune background. **a** Antigenic map of SARS-CoV-2 variants using plasma from vaccinated and/or breakthrough infected individuals. SARS-CoV-2 variants are depicted as circles, while plasma types are shown as squares. Each square represents an individual’s NT_50_ of the plasma, color-coded by the infecting variant. Both map axes represent antigenic distance; each grid square (1 antigenic unit) indicates a 2-fold change in neutralization titer. The proximity of points reflects antigenic similarity: closer points suggest higher cross-neutralization and greater antigenic likeness. Antigenic distance between WT and BA.1, BA.2 or BA.5 in the plasma of I-I-I (**b**), I-I-M (**c**), and M-M-M (**d**) vaccinated groups. Antigenic distance between WT and BA.1, BA.2, BA.5 or XBB.1.9.1 in the plasma of I-I-D (**e**), I-I-O (**f**) and M-M-O (**g**) vaccinated and subsequently breakthrough infected individuals with Delta and early Omicron subvariants. Antigenic distance between WT and BA.1, BA.2, BA.5, or XBB.1.9.1 in the plasma of I-I-I-B5 (**h**) and A-A-B5 (**i**) vaccinated and BA.5 breakthrough infected individuals

**Fig. 3:**
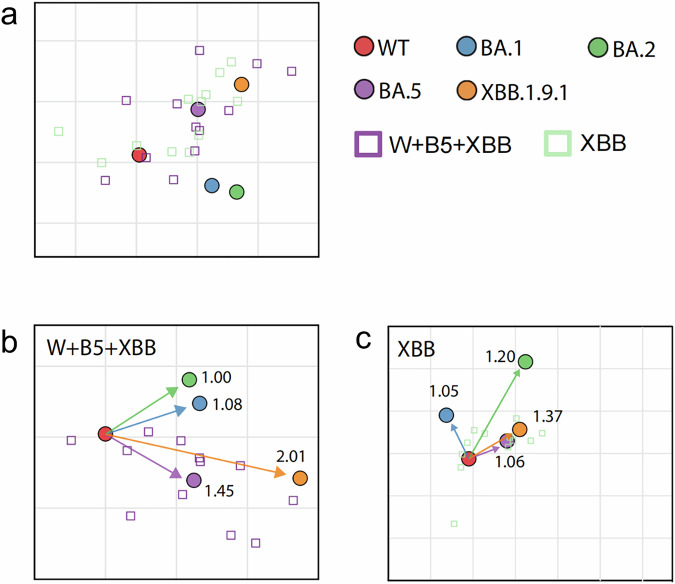
Antigenic cartography analysis in WT-vaccinated, BA.5-breakthrough infected, and XBB.1.5 vaccinated plasma. **a** Antigenic map of SARS-CoV-2 WT and variants based on differently XBB.1.5-targeted vaccination post-BA.5 infection. SARS-CoV-2 variants are shown as circles and plasma types are indicated as squares. Antigenic distance between WT and BA.1, BA.2, BA.5 or XBB.1.9.1 in the plasma of I-I-B5-(W + B5 + XBB) (**b**) and I-I-B5-XBB (**c**)

Large-scale vaccination or widespread infection has great impact on immune imprints of the human population. Several research evidence supports the notion that in populations with a specific background of vaccine-induced immunity or prior infection, the perceived antigenic distance between different viral strains may be shorter compared to the natural antigenic distance (the inherent antigenic difference observed in a naive population with no prior immune exposure).^15–17^ To evaluate antigenic divergence across vaccination/infection histories, we constructed antigenic maps using a previously validated multidimensional scaling (MDS) algorithm [6], based on NAb titers against WT, BA.1, BA.2, and XBB.1.9.1 variants. For each sample, twofold change of NT_50_ corresponds 1 unit of antigenic distance. The antigenic distance of the group was obtained by average of the fold changes of all samples. Antigenic cartography revealed that antigenic distance between WT and BA.1 or BA.2 was similar in all groups investigated (Fig. 2). While the distance from WT to BA.5 maintained high in prototype vaccinated groups (Fig. 2b–d) and the Delta breakthrough infected group (Fig. 2e), but decreased sharply from I-I-O onwards (Fig. 2f–i). Notably, the antigenic distance of XBB.1.9.1 exhibited the maximal separation from the WT strain (Fig. 2). For comparison, the ratios of NAb titers of WT/BA.5 were generated, and summarized with BA.5 and XBB.1.9.1 antigenic distance from WT in Table 1. Results show that primary vaccination cohorts (I-I-I, I-I-M, M-M-M) and WT-primed, Delta breakthrough infected individuals (I-I-D) maintained elevated WT:BA.5 NAb ratios (10.2-18.9), consistent with their longer BA.5 antigenic distances (3.35-4.24; Fig. 2b–e and Table 1). Whereas, Omicron breakthrough infections (I-I-O [BA.1/2], M-M-O [BA.1/2], I-I-I-B5, A-A-B5) significantly reduced WT:BA.5 ratios (1.8–4.4) while narrowing BA.5 antigenic distances (1.06–2.26) and expanding XBB.1.9.1 distances (3.32–5.03; Fig. 2f–i and Table 1). Strikingly, sequential BA.5 → XBB exposures (vaccination post-BA.5 infection) further diminished WT:BA.5 ratios to 0.4-1.0, accompanied by minimal BA.5 (1.06–1.45) and XBB (1.37–2.01) antigenic distances (Fig. 3 and Table 1). Collectively, the distance between WT and BA.5 decreased sharply from I-I-O onward, and the distance between WT and XBB dropped significantly at XBB.1.5 targeted vaccination group.

**Table 1:** Summary of WT/BA.5 ratios and antigenic cartography

Group/immune imprinting index	WT/BA.5 (ratio)	BA.5 antigenic distance	XBB antigenic distance
I-I-I, I-I-M, M-M-M, I-I-D	10.2–18.9	3.35–4.24	Not available
			I-I-D: 6.59
I-I-O, M-M-O, I-I-I-B5, A-A-B5	1.8–4.4	1.06–2.26	3.32–5.03
I-I-B5-XBB, I-I-B5-(W + B5 + XBB)	0.4–1.0	1.06–1.45	1.37–2.01

### XBB vaccination redirects immune imprinting from ancestral to Omicron variants and subverts antigenic seniority

**Fig. 4:**
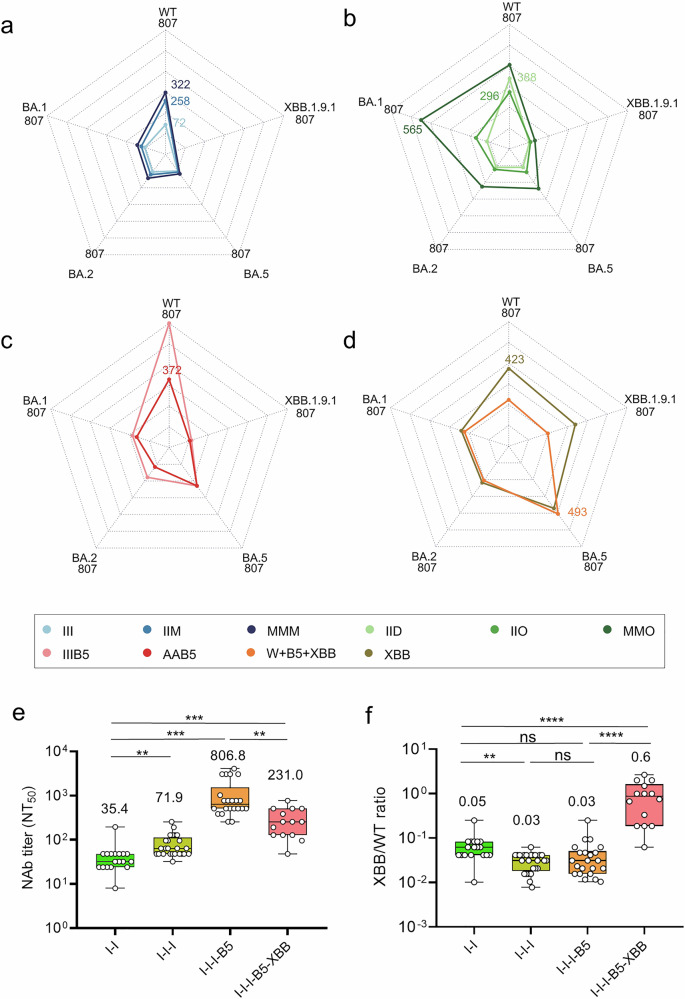
Immune trajectory from WT toward XBB.1.9.1 at different hybrid immune background. Radar plot analysis of the median neutralization titer against different variants in WT-vaccinated groups (**a**: I-I-I, I-I-M, M-M-M), vaccinated and Delta/early Omicron breakthrough infected groups (**b**: I-I-D, I-I-O, M-M-O), vaccinated and BA.5 breakthrough infected groups (**c**: I-I-I-B5, A-A-B5), and vaccinated, BA.5 infected, and subsequently XBB-targeting vaccinated groups (**d**: I-I-B5-[W + B5 + XBB], I-I-B5-XBB). **e** Dynamic of NAbs against WT in the prototype vaccinated (I-I, I-I-I), vaccinated followed by BA.5 infected (I-I-I-B5) and vaccinated followed by consecutive BA.5 and XBB.1.9.1 infected (I-I-I-B5-XBB) individuals. **f** Ratios of XBB versus WT NAb titers at the above groups of (**e**). Data are presented as mean ± SEM. Numbers above the dot plots and bars are geometric mean titers (**e**) or geometric mean foldchanges (**f**) with *p* value as indicated (Student’s *t* test, **: *p* < 0.01, ***: *p* < 0.001, ****: *p* < 0.0001)

Classical antigenic seniority posits that initial immune exposures dominate subsequent antibody responses, constraining adaptation to antigenically distant variants. Immune imprinting shift bias toward initial WT antigen exposure, directly impacts COVID-19 vaccine strain selection, immunization strategies, and guides priority population to get booster immunization. In order to get an overall perspective on immune imprinting shift by visualizing the trends of immune imprinting in a more intuitive manner, the migration of immune seniority was analyzed by radar plots (Fig. 4). Radar plot charts show that the immune radar pattern changed gradually and the center moved dynamically from WT to XBB in serial groups of different vaccination and consecutive infections. While prototype vaccination (I-I-I, M-M-M) and early Omicron breakthrough infections (I-I-D, I-I-O) exhibited persistent WT-centric antibody profiles (Fig. 4a–c), sequential BA.5 → XBB immune exposures through XBB.1.5-targeted vaccination fundamentally reconfigured humoral immunity. Primary vaccination groups, three-dose inactivated (I-I-I), mRNA (M-M-M), and heterologous mRNA-boosted (I-I-M) regimens, exhibited antibody profiles predominantly centered on the WT strain (Fig. 4a). This WT-centric imprinting persisted in Delta breakthrough infections (I-I-D; from September to November 2021), which elicited minimal Omicron-specific NAbs despite maintaining robust WT responses (Fig. 4b). A paradigm shift emerged during Omicron BA.1/BA.2 breakthrough waves (from December 2021 to January 2022): I-I-O cases demonstrated progressive antibody redirection from WT toward BA.1/BA.2, with mRNA-vaccinated followed by BA.1 breakthroughs (M-M-O) exhibiting stronger bias toward BA.1 (Fig. 4b). Intriguingly, BA.5 breakthroughs post-inactivated vaccination (I-I-I-B5) paradoxically elevated WT-specific NAbs while broadening reactivity against BA.1/BA.2/BA.5 beyond Delta or early Omicron exposures (Fig. 4c), suggesting dynamic imprinting evolution from WT dominance toward Omicron subvariants through successive immune exposures.

Critically, XBB.1.5-targeted vaccination after BA.5 infection (monovalent or trivalent recombinant protein vaccines) fundamentally reconfigured neutralization profiles, shifting the epicenters to BA.5 and XBB.1.9.1 (Fig. 4d). Radar plot analyses revealed a revolutionary shift that neutralization epicenter shifted from WT to BA.5 or XBB.1.9.1. Additionally, the WT-specific NAb titers gradually climbed from two-dose inactivated vaccination (I-I), to three-dose inactivated vaccination (I-I-I) until vaccinated and breakthrough infection with BA.5 (I-I-I-B5, NT_50_ = 807), while the titers of three inactivated vaccination followed by sequential BA.5 and XBB breakthrough infection (I-I-I-B5-XBB) dramatically decreased about 4-fold compared to I-I-I-B5 (Fig. 4e). Consistently, the ratio of XBB.1.9.1/WT NAbs maintained at similar levels (0.03–0.05) until I-I-I-B5, while sharply elevated to 0.6 at the I-I-I-B5-XBB group (Fig. 4f). These data conclusively demonstrate that XBB-targeted immunization marks a watershed transition in population immunity from ancestral virus imprinting to Omicron-adapted antibody landscapes, suggesting that sufficiently antigenic divergent variants can overcome the original antigenic seniority through structural epitope refocusing rather than incremental drift. Our findings establish XBB-lineage antigens as critical compartments for overcoming historical immune imprinting, which is of great significance for pandemic preparedness.

### Quantitative modeling of XBB.1.9.1 protection threshold

**Fig. 5:**
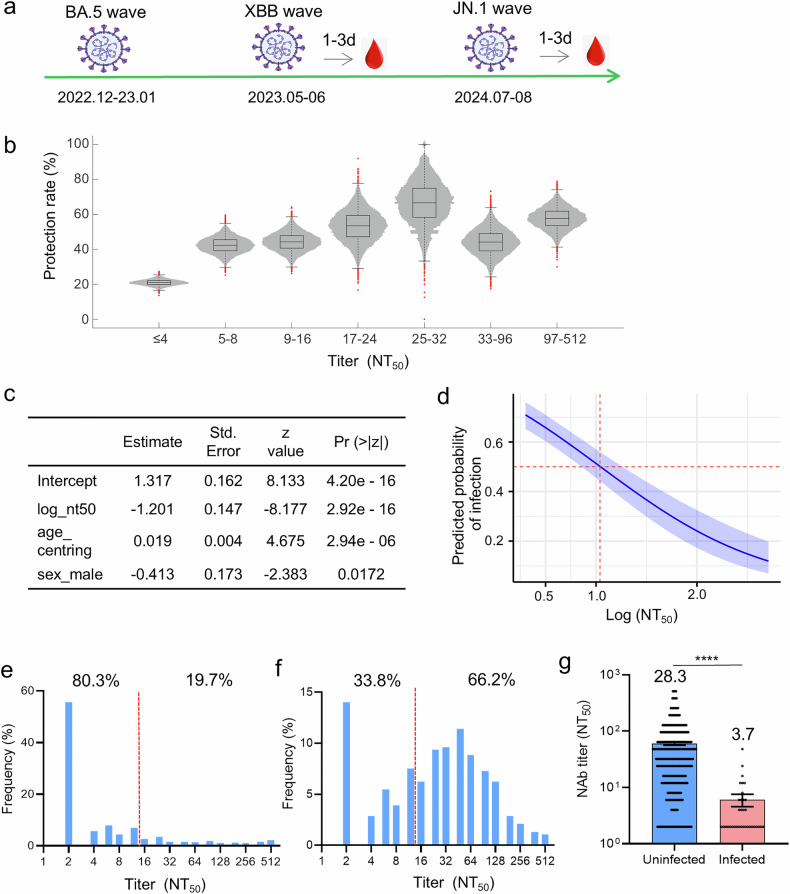
The association between protection and NAb titers. **a** Schematic for schedule of sampling. **b** The protection rate at different scale of NAbs. A violin plot showing the distribution of the protection rate for different groups of people stratified by their antibody titer for the XBB.1.9.1 strain. Each distribution for a single group contains 10,000 samples. The box plots superimposed show the interquartile range (IQR) with the median (the boxes), 1.5 times the IQR (the whiskers) and the outliers (red dots). **c** Coefficients of multivariable logistic regression analysis. **d** Adjusted association curve between NT_50_ titer and predicted infection probability. The model was conditioned on median age and baseline gender (female). The solid blue line represents the point estimates of predicted probability, while the blue shaded area denotes the 95% confidence interval derived from the standard error of predictions based on the logistic regression model. The horizontal dashed red line indicates the 50% probability threshold, and the vertical dashed red line shows the estimated NT_50_ titer value corresponding to a 50% predicted infection risk for individuals with the reference characteristics (median age and female gender). The narrow confidence interval around the curve indicates that the estimation of the dose-response relationship has high precision across the entire range of antibody titers. **e** XBB.1.9.1 NAb titer distribution of cohort collected from fever clinic in May-June of 2023 (XBB endemic), and the dashed red line indicates the 50% protection threshold. **f** JN.1 NAb titer distribution in cohort collected from fever clinic in August of 2024 (JN.1 endemic), and the dashed red line indicates the 50% protection threshold. **g** NAb titers against JN.1 at plasma of uninfected and infected individuals at JN.1 endemic. Data are presented as mean ± SEM. Student’s *t* test was performed for the statistics analysis between uninfected and infected geometric mean titers with *p*-value as indicated (****: *p* < 0.0001)

To investigate the protective threshold, a total of 926 febrile patients were recruited from the Fever Clinic of Guangzhou First People’s Hospital, following the timeline illustrated in Fig. 5a. The NAb titers were tested, and the temporal alignment allowed us to model the relationship between protection and baseline NAb titers of the population. Firstly, the protective rate, representing the proportion of un-infected individuals accounting for the total number at different antibody titer range were calculated. Using bootstrapping method, the distribution of protective rate across different stratified NAb titers was visualized by violin plots. The association of protection rate versus the NAb titer clearly shows that the NAb titer was positively correlated to the protection rate (Fig. 5b).

To calculate the protective threshold of XBB-specific neutralizing antibody in the population, we employed multivariable logistic regression analysis to construct a model, in which the association between antibody titer and infection risk was quantified. The regression coefficients of the model were estimated using the maximum likelihood method. Among the independent variables, NAbs emerged as a significant negative predictor of XBB infection (*β* = −1.201, *p* < 0.001). In contrast, age was found to be significantly positively associated with infection risk (*β* = 0.019, *p* < 0.001) (Fig. 5c). Overall, among the variables examined, NAbs represented the strongest protective factor against XBB infection, with its effect magnitude being substantially greater than those of age and gender. Using the multivariable logistic regression model, an adjusted association curve between NAbs and predicted infection probability was generated (Fig. 5d). The curve illustrates the relationship between NT_50_ (log10 scale) and predicted infection probability. A strong, non-linear negative correlation was observed: higher antibody titers were associated with lower predicted infection risk. The infection risk gradient was steepest at lower titer levels and tended to flatten out at higher titer levels. The vertical dashed line represents the estimated NAb value corresponding to a 50% predicted infection risk for individuals with the reference characteristics (median age and female sex). The 50% protective threshold was estimated to be NT_50_ = 12.6 (95% CI 7.2–18.6) (Fig. 5d).

Stratification of XBB.1.9.1-specific NAb titers across 926 individuals revealed stark contrasts: 55.6% (515/926) exhibited titers below the detection limit (NT_50_ = 4), among which, 65.7% (417/635) of the PCR-positive COVID-19 cases showed NAb titers that below the detectable XBB.1.9.1-specific NAbs (NT_50_ = 4), compared to that of 33.7% (98/291) in PCR-negative group. This dichotomy suggests the immunity from prior exposures provided limited protection against XBB.1.9.1 (Table 2).

**Table 2:** Distribution of NAb titers in SARS-CoV-2 ± individuals

Titer range	Total (*n*)	Total (%)	SAR-CoV-2 (−) (*n*)	SAR-CoV-2(−) (%)	SAR-CoV-2 (+) (*n*)	SAR-CoV-2 (+) (%)
<4	515	55.6	98	33.7	417	65.7
4–16	253	27.3	108	37.1	145	22.8
17–32	47	5.1	27	9.3	20	3.2
33–96	45	4.9	20	6.9	25	3.9
>96	66	7.1	38	13.0	28	4.4

Population-level analysis revealed that 80.3% (744/926) of individuals had XBB.1.9.1 titers below the protective threshold (NT_50_ = 12.6), aligning with the observed 68.6% (635/926) infection prevalence at the investigated stage (Fig. 5e and Table 2). Strikingly, subsequent JN.1 variant surveillance in July to August 2024 demonstrated threshold validity: 66.2% (255/385) of individuals maintained NAb titers above 12.6 against the circulating subvariant JN.1 (Fig. 5f), coinciding with much lower infection rate (9.1%, 35/385) in febrile populations. The geometric mean titer in JN.1-infected cases (3.7, 95% CI: 2.8–5.0) was more than sevenfold lower than uninfected controls (28.3, 95% CI: 24.5–32.6; Fig. 5g), reinforcing the general applicability of this threshold across evolving Omicron subvariants.

## Discussion

The progressive evolution of SARS-CoV-2 has necessitated a paradigm shift in understanding how hybrid immunity induced by iterative vaccination and infection reshapes population-level antibody landscapes. Our study provides critical insights into two interconnected phenomena: (1) the dynamic migration of immune imprinting from ancestral virus-centricity to Omicron subvariant dominance, and (2) the establishment of the first quantitative protection threshold against XBB subvariants, a cornerstone for rational pandemic preparedness. This discussion will also incorporate the concepts of antigenic distance, the original antigenic sin and serotype classification to offer a more comprehensive discussion.

The traditional concept of “natural antigenic distance”, derived from inherent, genetically and structurally determined degree of difference between antigens (typically key immunogenic molecules like the spike protein of SARS-CoV-2) derived from distinct strains of a pathogen. Antigenic distance is usually measured under the premise of evaluating immune-naive systems using sera from individuals with no prior contact with the pathogen or in animal models with no pre-existing pathogen-specific immunity. However, emerging evidence strongly suggests that pre-existing population immunity, sculpted by vaccination and prior infection, functionally alters this perceived distance, often effectively shortening it. The key mechanism is the induction of cross-reactive memory B-cells and T-cells targeting conserved epitopes shared across variants. Our observations on that the prototype vaccination followed by BA.1/BA.2/BA.5 infection (I-I-O, M-M-O, I-I-I-B5, and A-A-B5) significantly shortened the antigenic distance of BA.5 to WT (Fig. 2) and XBB.1.5-targeted vaccination of the BA.5 infected individuals (I-I-B5-XBB) or (I-I-[W + B5 + XBB]) clearly shortened the antigenic distance of XBB to WT (Fig. 3) strongly support the notion. Understanding the antigenic distance in the context of the specific immune background of a population is conducive to selecting suitable vaccine strains and enhancing protective efficacy. Furthermore, for populations with different immune backgrounds, antigenic distance analysis enables the identification of priority groups for vaccination and the adoption of more targeted special immunization procedures, such as sequential vaccination.

Classical antigenic seniority posits that primary immune exposures dominate subsequent antibody responses, a phenomenon known as original antigenic sin. In the context of SARS-CoV-2, our data challenge this traditional view. Initial prototype vaccinations and infections with early variants like Delta BA.1 and BA.2 led to a dominant WT-centered immune response, which aligns with the concept of original antigenic sin.^18^ However, exposure to sufficiently divergent Omicron subvariants, especially XBB lineages, can override the ancestral virus immune imprinting. The transition from WT-centric responses in prototype-vaccinated cohorts to BA.5/XBB-dominated profiles in XBB post-boosting individuals is significant (Fig. 4). This “immune reset” mechanism is distinct from incremental antigenic drift. Furthermore, XBB-targeted vaccination was more effective than natural BA.5 breakthrough infection in achieving a durable redirection of the neutralization focus. This indicates that engineered antigens can be more efficient in overcoming historical immune imprints, highlighting the importance of strategic vaccine design in the face of viral evolution.

The dynamic changes in immune imprinting are also closely related to SARS-CoV-2 serotype classification. Recent studies propose classifying SARS-CoV-2 into serotypes based on the humoral immune response and surface antigens. For example, the ancestral virus and main variants like Alpha, Beta, Gamma, and Delta may be considered as serotype 1, while Omicron sublineages such as BA.1, BA.2, and BA.3 could be serotype 2 due to their significant genetic and antigenic differences.^19^ A later study demonstrated that the virus can be classified into five serotypes, including subtype I (Prototype until Kappa subvariant), subtype II (BA.1 and subvariants), subtype III (BA.2 and subvariants and BA.3), subtype IV (BA.5, BF.7, and BQ1 and subvariants), and subtype V (XBB, XBB.1.5 and subvariants), based on systematic evaluation of the antigenicities of RBD.^20^ Our study shows that the immune imprinting gradually shifts from WT to XBB subvariants after sequential exposure to BA.5 and XBB.1.5 antigens through infection and vaccination, respectively (Fig. 4). Based on the concept that different serotypes can evoke distinct immune responses, the unique antigenic properties of XBB subvariants may indicate that they could be part of a new serotype or a distinct antigenic cluster within the Omicron related serotype. Their ability to override the original antigenic imprinting related to the WT strain further emphasizes their antigenic divergence, which has implications for serotype-based immune protection.

The changes in the dominant centers of population immunity have a direct impact on vaccination strategies. The vaccination targeting the WT strain together with the nationwide pandemic of BA.5 between the end of 2022 and early 2023 had established a basic level of immunity in the Chinese population. However, with the emergence of new variants like XBB, the original immune dominance needed re-evaluation. In the small cohort of study, we showed that after XBB-targeted vaccination, the immune response became more focused on XBB subvariants (Fig. 4). Additionally, the XBB reinfection after BA.5 have also greatly increased the ratio of XBB/WT NAbs. They imply that boosting with XBB targeted vaccines sufficiently induced profound NAbs against divergent of XBB antigen in the context of specific immune background. In terms of serotypes, future vaccines should be designed to target the currently dominant and emerging serotype-associated variants. Booster vaccinations should be scheduled in accordance with the changing immune status of the population and the prevalence of different serotypes. When a new variant, like XBB, with high immune escape capabilities emerges, a timely booster shot that can enhance the immune response against this variant and associated serotype compartments is essential.

Several studies have shown that higher levels of neutralizing antibody are associated with immune protection from symptomatic SARS-CoV-2 infection during short-term follow-up after vaccination.^21,22^ These studies also tried to estimate the level of protection associated with particular antibody levels to estimate the relationship between neutralizing antibody levels and vaccine efficacy. While neutralizing antibody levels are a clear CoP, identifying a “protective threshold” applicable to a serological test is more challenging. In terms of a protection “threshold”, there have been a few studies estimated that the neutralizing antibody level associated with 50% protection from COVID-19 was ≈20% of the mean titer for persons in the convalescent phase (or 54 IU/mL), and 70% protective thresholds ranging from 4 to 33 IU/mL.^21^ However, these studies have been based on protection of vaccination against prototype virus or virus before Delta strains. Protection based on the immune memory from vaccination and previous infection against XBB strains have not been reported. Furthermore, previous studies on the protective threshold of COVID-19 have all been based on clinical investigation research following vaccine-induced immunity, which is thus highly dependent on vaccine utilization or clinical trials. This section of the present study did not consider the influence of vaccine-induced immunity or infection history, and instead focused on establishing a model to calculate the protective threshold by measuring NAbs in populations with and without COVID-19 infection during an infection wave. It thereby provides an alternative solution for the research on the COVID-19 protective threshold. In the present study, we identified an NT_50_ of 12.6 as the 50% protective threshold against XBB.1.9.1, filling a critical gap in Omicron vaccinology. This threshold aligns with the findings of a study performed with murine challenge models but is lower than ancestral-virus-based estimates.^23^ Retrospective analysis showed that in mid-2023, 80.3% of the population fell below this threshold, coinciding with the XBB resurgence (infection rate: 68.6%), while the minority of the population had sub-protective JN.1 titers by August 2024, explaining the limited spread (infection rate: 9.1%) of JN.1 variant. Our findings suggest that if a significant portion of the population has NAb titers lower than the 50% protective threshold against newly emergent or the circulating variants or serotype-related compartments, it may be necessary to accelerate the rollout of booster vaccinations or develop new vaccines specifically targeting the antigenic entity. Determining the protective threshold for SARS-CoV-2 provides a quantifiable standard for immune protection, shifting the assessment of individual or population immune levels from vague judgment to precise measurement. It guides clinical individualized immune interventions and public health monitoring, help identifying high-risk groups, warns of epidemic risks, and optimize prevention strategies.

In conclusion, combined with the concepts of serotype classification and original antigenic sin, our study provides a more in-depth understanding of the immune response to SARS-CoV-2. These findings have important implications for optimizing vaccine strategies, predicting the risk of emerging infections, and controlling the spread of the virus. Our future research will focus on validating these results in larger populations. Exploring the long-term durability of the immune response against emerging variants within the serotype framework is crucial. Additionally, understanding how different immune imprinting patterns interact with novel vaccine platforms, such as mRNA-based or vector-based vaccines, could lead to more tailored and efficient vaccination strategies. It is also essential to continue researching the antigenic differences between XBB subvariants and other serotype-associated variants to refine serotype classification and improve vaccine design.

## Materials and methods

### Ethics statement

This study is approved by the Ethics Commission of the First Affiliated Hospital of Guangzhou Medical University (No.2021-78). Written informed consent was obtained from all patients and volunteers.

### Study design and cohort demographics

To investigate dynamic immune responses in subpopulations with heterogeneous immune histories over the past years, we recruited participants based on their vaccination and infection timelines, categorizing them into four distinct groups, with their demographics shown in Table 3:

1. Group I: individuals who received prototype-based vaccination.
2. Group II: prototype-vaccinated individuals breakthrough infected with Delta strains or early Omicron subvariants (BA.1/BA.2).
3. Group III: prototype-vaccinated individuals who experienced BA.5 breakthrough infection.
4. Group IV: prototype-vaccinated and BA.5-infected individuals who subsequently vaccinated with XBB.1.5-targeted vaccine.

The vaccination groups included individuals receiving inactivated viral vaccines (two-dose [I-I] or three-dose [I-I-I] regimens), three-dose mRNA vaccines (M-M-M), or heterologous booster regimens (two inactivated doses followed by an mRNA vaccination [I-I-M]). The breakthrough infection groups comprised: (a) recipients of two inactivated vaccine doses followed by subsequent Delta (I-I-D) or Omicron BA.1/2 (I-I-O) breakthrough infections, as well as three inactivated doses followed by BA.5 (I-I-I-B5) breakthrough infections; (b) mRNA vaccine recipients (two doses of BNT162b2 or mRNA-1273) experiencing Omicron BA.1/2 breakthroughs (M-M-O); and (c) recipients of two doses adenovirus vectored vaccine followed by BA.5 breakthrough infections (A-A-B5). The XBB-targeted vaccination post-BA.5 infection groups encompassed individuals receiving WT + BA.5 + XBB.1.5 multivalent (I-I-B5-[W + B5 + XBB]) or XBB.1.5 monovalent (I-I-B5-XBB) recombinant protein vaccines after BA.5 infection.

**Table 3:** Vaccination and infection cohort demographics

Cohort	Sample size (*n*)	Gender		Age		dpv/dpi*
		Male	Female	0–59	>59	
I: I-I	35	21 (60.0%)	14 (40.0%)	35 (100%)	0 (0%)	28
I: I-I-I	30	16 (55.3%)	14 (46.7%)	30 (100%)	0 (0%)	28
I: I-I-M	21	6 (28.56%)	15 (71.4%)	16 (76.2%)	5 (23.8%)	28
I: M-M-M	25	11 (44.0%)	14 (56.0%)	19 (76.0%)	6 (24.0%)	28
II: I-I-D	22	15 (68.2%)	7 (31.8%)	22 (95.7%)	0 (0%)	28
II: I-I-O	23	18 (78.3%)	5 (21.7%)	22 (95.7%)	1 (4.4%)	28
II: M-M-O	19	10 (52.6%)	9 (47.4%)	17 (89.5%)	2 (10.5%)	28
III: I-I-I-B5	23	7 (30.4%)	16 (69.6%)	23 (100%)	0 (0%)	28
III: A-A-B5	44	24 (54.5%)	20 (45.5%)	32 (72.7%)	12 (27.3%)	28
IV: I-I-B5-(W + B5 + XBB)	12	7 (58.3%)	5 (41.7%)	12 (100%)	0 (0%)	28
IV: I-I-B5-XBB	12	8 (66.7%)	4 (33.3%)	12 (100%)	0 (0%)	28

^*^dpv days post vaccination, *dpi* days post infection

From 2020 to the end of 2022, regular nucleic acid amplification testing (NAAT) for SARS-CoV-2 was implemented in China due to the lockdown policy. This practice ensured that the SARS-CoV-2 infection history of the population was well-documented and traceable. For the different vaccinated groups (I-I-I, I-I-M, and M-M-M), participants were enrolled only after completing the corresponding vaccination regimen and were confirmed to have no prior history of SARS-CoV-2 infection. For the I-I-D, I-I-O and M-M-O groups, samples were collected from hospital-based individuals, whose SARS-CoV-2 infection had been laboratory-confirmed via NAAT. The BA.5 breakthrough infection cohorts (I-I-I-B5 and A-A-B5) were recruited during the SARS-CoV-2 BA.5/BF.7 variant wave in China, which occurred from the end of 2022 to early 2023. All samples in this cohort were collected from southern China, and genetic characterization confirmed that the predominant infecting variant was SARS-CoV-2 BA.5. The XBB.1.5-targeted vaccination groups were enrolled after one shot of multivalent or monovalent recombinant protein vaccination with prior two-dose inactivated viral vaccination and BA.5 breakthrough infection (Fig. 1a).

### Study design for XBB protection threshold analysis

To investigate the neutralizing antibody protective threshold against XBB subvariant infection, we recruited 926 febrile patients from the fever clinic at Guangzhou First People’s Hospital. Nucleic acid testing identified 635 individuals (68.6%) as SARS-CoV-2 positive and 291 (31.4%) to be negative. The cohort comprised 42.3% males and 57.7% females, with 33.9% aged >59 years (Table 4). All participants presented with fever (≥38 °C), and plasma samples were collected at their initial hospital visit.

**Table 4:** Demographics of fever clinic cohort in May–June 2023

	Total	SARS-CoV-2 (−)	SARS-CoV-2 (+)
Gender			
Male	392 (42.3%)	132 (14.2%)	260 (28.1%)
Female	534 (57.7%)	159 (17.2%)	375 (40.5%)
Age			
0-17	67 (7.2%)	24 (2.6%)	43 (4.6%)
18-59	545 (58.9%)	190 (20.5%)	355 (38.4%)
>59	314 (33.9%)	77 (8.3%)	237 (25.6%)

Given the reported 1- to 3-day incubation period for Omicron variants, these samples obtained at fever onset were assumed to represent early infection stage (≤3 days post-XBB exposure). During this phase, the NAb titers remained comparable to pre-infection levels due to insufficient time for de novo immune responses. This temporal alignment allowed us to model the relationship between baseline NAb titers (measured in all subjects) and infection status, thereby estimating the 50% protective threshold against XBB infection.

### Plasma sample collection for threshold validation

To validate the protective antibody threshold, plasma samples were collected from 385 febrile patients at the Fever Clinic of Guangzhou First People’s Hospital from July to August 2024, during the time when JN.1 had become the dominant circulating subvariant. SARS-CoV-2 infection status was determined through NAAT and/or nucleocapsid protein (NP) antigen detection. Of the enrolled participants, 35 (9.1%) were tested SARS-CoV-2 positive while 350 (90.9%) were tested SARS-CoV-2 negative. The demographic data were summarized in Table 5.

**Table 5:** Demographics of fever clinic cohort in July-August 2024

	Total	SARS-CoV-2 (−)	SARS-CoV-2 (+)
Gender			
Male	178 (46.2%)	163 (42.3%)	15 (3.9%)
Female	207 (53.8%)	187(48.6%)	20 (5.2%)
Age			
0-17	21 (5.5%)	21 (5.5%)	0 (0.0%)
18-59	264 (68.5%)	250 (64.9%)	14 (3.6%)
>59	100 (26.0%)	79 (20.5%)	21 (5.5%)

### Authentic SARS-CoV-2 virus microneutralization assay

The NAbs in plasma against SARS-CoV-2 prototype and different variants were determined by using a cytopathic effect (CPE)-based microneutralization assay as previously described.^24^ The virus strains and variants used include: the wild-type strain (GenBank: MT123291), and Omicron variant, including subvariant BA.1 (IQTC-Y216017), BA.2 (IQTC-IM22003633), BA.5 (GDCPP.2.00303), XBB.1.9.1 (IQTC-IM2396943), and JN.1 (IQTC-IM2423535). The BA.5 subvariant was isolated at Guangzhou Center for Disease Control and Prevention. The rest of variants were isolated at Guangzhou Customs Technology Center, Guangzhou, China. In brief, two-fold serial dilutions (1:4-1:512 or 1:2-1:256) of heat-inactivated plasma were tested in duplicate wells for the presence of NAbs in monolayer of Vero E6 cells. 100 TCID_50_ of virus in 50 μl/well was incubated with 50 μl of plasma in 96-well plates for 2 h. Vero E6 cells were trypsinized and resuspended in Dulbecco’s Modified Eagle Medium (DMEM) containing 4% of fetal bovine serum and 1% of pen/strep at a concentration of 1.2 × 10^5^ cells/ml and 100 μl of cell suspension were then added into the 96-well plates, followed by incubation at 37 °C, 5% CO_2_ for 4 days. The neutralization was determined by the appearance of CPE in images captured with Celigo Image Cytometer (Revvity, formerly known as Nexcelom Bioscience, Lawrence, MA). The NT_50_ was defined as the reciprocal of the highest sample dilution that protected 50% of wells from CPE.

To guarantee the assurance of the data, rigorous quality control (QC) measures were implemented throughout the study. Firstly, all the assays were performed according to a highly standardized operation procedure. Secondly, we’ve developed positive controls for calibrating our detection system and ensuring the comparability and credibility of the data. Thirdly, three QCs, including positive control, negative control and virus back-titration, were incorporated into each microneutralization assay. The experiment was deemed valid only when the results of all three QC measures fall within their respective calibrated ranges.

### Association analysis of protection rate and neutralization titers

#### Calculation of the protection rate stratified by the level of antibody titer

For a set of data recording the infection status (i.e., positive or negative) and antibody titer for each person in a cohort, we stratified the cohort into seven groups based on their antibody titers (e.g., less than 4, 5–8, 9–16, 17–24, 25–32, 33–96, 97–512) and the protection rate for each group was the percentage of people with a negative test outcome in the group.

#### Estimation of the uncertainty of the protection rate

We used the bootstrapping method to resample from the original data set in order to quantify the uncertainty of the protection rate for each group. In detail, suppose the data set include infection status and antibody titer for K people. To generate 10,000 samples of protection rate for each group, the method takes the following steps: a. K samples from the data set were randomly selected (with replacement), b. K samples were stratified into the seven groups specified above based on the antibody titer, c. The protection rate was calculated for each of the seven groups, d. The step 1–3 were repeated for 10,000 times. After the step d, 10,000 samples of the protection rate for each of the groups were obtained. The distribution of the protection rate for each group was then visualized by a violin plot.

### The protective threshold modeling based on NT50 against XBB.1.9.1

To quantitatively assess the protective effect of antibody level against XBB.1.9.1, we generate protection curve based on a hypothesis that the antibody titer may provide protective effect when it’s above a certain threshold. The model was generated by taking the data, including infection status, NT_50_ values, and demographic variables into consideration. The NAb titers that <4 were taken as 2. We fitted a multivariable logistic regression model with XBB infection status as the dependent variable (1 = positive, 0 = negative). Independent variables included log10-transformed NT_50_, age (centered and modeled as a continuous variable), and sex (treated as a categorical factor). The 50% protective threshold (PT_50_) was defined as the NT_50_ value corresponding to a predicted infection probability of 0.5. To account for covariates, a conditional threshold was estimated at predefined reference values (sex = female, age = sample median). We further quantified the proportion of individuals with NT_50_ values below PT_50_ and estimated the corresponding confidence interval using bootstrap resampling. Finally, the NT_50_ at 50% of the protective rate was calculated.

### Antigenic cartography

Antigenic distances between SARS-CoV-2 variants were estimated by integrating the neutralization potency of each serum sample using a previously described antigenic cartography method.^6^ In brief, an antigenic map was constructed using a set of NAbs in plasma against a panel of antigens. The titer table was converted into a distance table through the calculation of log₂ (titer/10), with each value then adjusted by subtracting a “column base”, defined as the maximum value within each column in this analysis. Both the *x*-axis and *y*-axis denote antigenic distance. Since only the relative positions of antigens and NAbs can be determined, the orientation of the map within these axes is arbitrary. The interval between grid lines corresponds to 1 unit of antigenic distance, which is equivalent to a twofold change of plasma NAbs in the titer table. Two units correspond to a fourfold value, and this relationship extends accordingly (three units correspond to an eightfold value, etc.).

The map was generated using the Racmacs package (https://acorg.github.io/Racmacs/, version 1.1.4) in R, with 2,000 optimization steps performed and the minimum column basis parameter set to “none”. The tableDistances function within the Racmacs package was utilized to compute antigenic distances, and the average distances from all plasma to each variant were adopted to represent the final distances. Antigenic maps were then generated by using the MDS algorithm. The WT was designated as the origin of the map; thus, the table distance for the WT was manually reset to zero by subtracting the WT value from each column.

### Radar plot

The geometric average value was calculated for each group of plasma samples. The neutralization potency of different plasma to each SARS-CoV-2 variant was present with radar plot by using the R packages “fmsb”.

## Data Availability

All data generated or analyzed during this study are included in this published article.
